# Correction to “Highly Efficient Supramolecular Photocatalyst for CO_2_ Reduction with Eight Carbon–Carbon Bonds between Ru(II) Photosensitizer and Re(I) Catalyst Unit”

**DOI:** 10.1021/acscatal.3c04613

**Published:** 2023-10-25

**Authors:** Kei Kamogawa, Antonio Santoro, Ambra M. Cancelliere, Yuushi Shimoda, Kiyoshi Miyata, Ken Onda, Fausto Puntoriero, Sebastiano Campagna, Yusuke Tamaki, Osamu Ishitani

Page 9029 and Table 1: In the published article, the units for the emission lifetimes of Ru complexes (μs) are not correct, which should be ns. These corrections do not affect the conclusions of the paper at all.

**Table 1:** Photophysical Properties

	λ_em_/nm^a^	τ/ns^b^	*k*_q_^c^/10^8^ M^–1^ s^–1^	η_q_^d^
**RuC2PhC2Re**	641	761 ± 1	10	0.99
**RuC2Re**	644	762 ± 1	10	0.99
**Ru**	640	748 ± 1		

a Measured in DMA–TEOA (5:1 v/v) at 25 °C. The samples were saturated with CO_2_. The excitation wavelength: 460 nm.

b The excitation wavelength: 510 nm.

c The excitation wavelength: 520 nm.

d Quenching fraction with 0.1 M BIH: η_q_ = *k*_q_τ[BIH]/(1+*k*_q_τ[BIH]).

Page 9029:

Before correction: Because the emission lifetimes of **RuC2PhC2Re** (761 μs) and **RuC2Re** (762 μs) were close to that of **Ru** (748 μs) (Table 1), the intramolecular electron transfer from the excited Ru unit to the Re unit (i.e., oxidative quenching) was negligible for both **RuC2PhC2Re** and **RuC2Re**.

After correction: Because the emission lifetimes of **RuC2PhC2Re** (761 ns) and **RuC2Re** (762 ns) were close to that of **Ru** (748 ns) (Table 1), the intramolecular electron transfer from the excited Ru unit to the Re unit (i.e., oxidative quenching) was negligible for both **RuC2PhC2Re** and **RuC2Re**.

